# Spatially resolved *in vivo* plant metabolomics by laser ablation-based mass spectrometry imaging (MSI) techniques: LDI-MSI and LAESI

**DOI:** 10.3389/fpls.2015.00471

**Published:** 2015-07-10

**Authors:** Benjamin Bartels, Aleš Svatoš

**Affiliations:** Research Group Mass Spectrometry/Proteomics, Max Planck Institute for Chemical EcologyJena, Germany

**Keywords:** ambient, ionization, mass spectrometry, laser ablation, electrospray

## Abstract

This short review aims to summarize the current developments and applications of mass spectrometry-based methods for *in situ* profiling and imaging of plants with minimal or no sample pre-treatment or manipulation. Infrared-laser ablation electrospray ionization and UV-laser desorption/ionization methods are reviewed. The underlying mechanisms of the ionization techniques–namely, laser ablation of biological samples and electrospray ionization–as well as variations of the LAESI ion source for specific targets of interest are described.

## Introduction

Sample preparation is an important step that precedes acquisition of many kinds of data. However, often sample preparation is associated with artificially altering the biological or biochemical status of the system under study. In order to minimize this effect, we would like to have little to no sample preparation. If we can perform analysis directly *in vivo*, our data might fully represent the actual system. The usual workflow relies on sample dissection, solvent or thermal extraction and subsequent analysis using chromatographic methods connected to a detector with the needed selectivity. Minimal sample preparation facilitates the analytic process, by allowing people with minimal experience in analytical chemistry to perform the necessary steps without highly involved training. The sheer number of emerging ionization techniques involving minimal, ambient pressure sample preparation demonstrates the current interest, but, sadly, an alphabet soup of abbreviations has been created. Recent reviews (Bhardwaj and Hanley, 2014; El-Baba et al., 2014; Venter et al., 2014) summarize established techniques for most of the possible applications to date, providing an excellent guide for beginners to the field. These techniques are especially interesting for the life sciences (Alberici et al., 2010; Shrivas and Setou, 2012), due to the delicate nature of biological samples. Biological mass spectrometry imaging (MSI) is profoundly profiting from these developments.

**FIGURE 1:**
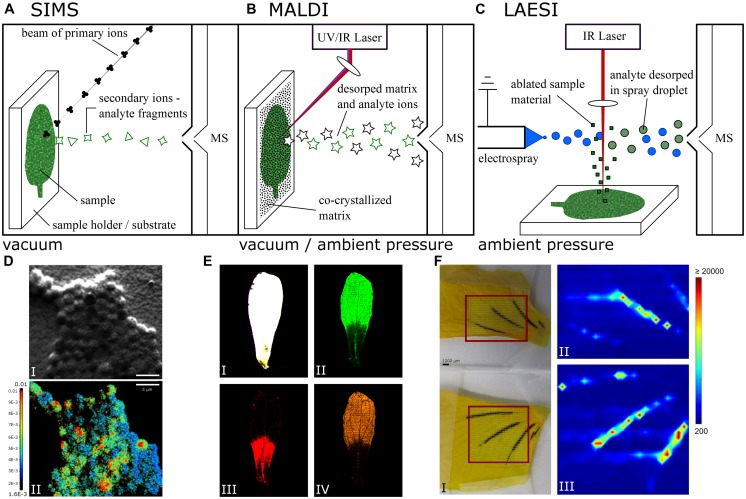
**Simplified schematic of **(A)** secondary ion mass spectrometry (SIMS), **(B)** matrix-assisted laser desorption/ionization (MALDI), and **(C)** laser ablation electrospray ionization (LAESI) mass spectrometry imaging (MSI). (D)** Color overlay showing the ^15^N/^14^N ratio measured with NanoSIMS (I) and the corresponding secondary electron image (II) of *Chorizanthe watsonii* cells, scale bars 5 μm. Adapted with permission from (Mohr et al., 2013). **(E)** Optical image (I) and color overlays of *Arabidopsis thaliana* petal LDI-MSI in negative ion mode corresponding to kaempferol (II), quercetin (III), and kaempferol rhamnoside (IV) with mass to charge ratios (*m/z*) of 285, 301, and 431, respectively. Adapted with permission from (Hölscher et al., 2009). **(F)** *Viola* petals (I) as sampled with conventional LAESI (top) and HA-LAESI (bottom). The color overlays II & III show the spatial distribution of the selected ion *m/z* 919.3 as sampled from the smaller petal with conventional LAESI (II) and from the bigger petal with HA-LAESI (III). Adapted with permission from (Vaikkinen et al., 2013). Copyright 2012 American Chemical Society.

In addition to being the least intrusive approach, spatial resolution is an important feature for any imaging technique. Secondary ion mass spectrometry (SIMS) is the ionization technique for mass spectrometry (MS) that offers highest spatial resolution down to reported values of below one micron (Svatos, 2010). Because it uses an ion beam to create secondary ions from the sample (**Figure 1A**), SIMS is not considered a soft ionization technique. Molecules tend to fragment upon ionization, and the utilization of SIMS is intrinsically linked to extensive sample preparation. SIMS has successfully been used on biological samples for imaging (McMahon et al., 1995). In 2013, SIMS was successfully used to investigate the dynamics of nitrogen gas fixation of cyanobacteria at the level of a single cell (Mohr et al., 2013; **Figure 1D**). MSI of intact biomolecules, however, struggles to reach the level of a bacterial cell. In contrast, recent advances report single-cell resolution on eukaryotes with matrix-assisted ionization techniques, involving extensive sample preparation prior to analysis (Boggio et al., 2011). In early 2015, single-cell imaging was done within a tissue (Li et al., 2015b) utilizing laser ablation electrospray ionization (LAESI), which requires considerably less sample preparation.

A prominent ionization technique used in MSI of large biomolecule imaging is matrix-assisted laser desorption/ionization (MALDI; Caprioli et al., 1997; Bjarnholt et al., 2014; El-Baba et al., 2014). MALDI instrumentation for MSI is commercially available with a spatial resolution of 10 μm (FLEX series, Bruker, Bremen, Germany). MALDI requires the samples to be pre-processed extensively by dissolution in and co-crystallization together with a matrix. Originally restricted to vacuum application (Feigl et al., 1983; Karas et al., 1985, 1987), MALDI has since been adapted to work under atmospheric pressure (Laiko et al., 2000; Li et al., 2007). Desorption and ionization of co-crystallized samples with matrix is facilitated by an ultraviolet (UV) laser and recently has also been used in conjunction with infrared (IR) lasers. The matrix molecules absorb most of the energy deposited to the sample by the laser and transfer the energy to the sample analytes more gently than via direct irradiation (Caprioli et al., 1997; Karas and Kruger, 2003), as depicted in **Figure 1B**. With MALDI, scientists can ionize very big molecules, e.g., proteins, non-destructively, which is one of the reasons why MALDI is used in protein MSI analysis. The method requires reliable matrix deposition and high ion yield (Karas and Kruger, 2003; El-Baba et al., 2014). To image plant cells – some as large as 50 μm – the spatial resolution of commercial instruments is sufficient. Laser desorption ionization (LDI) works similarly to MALDI but does not require an externally applied matrix. Because samples are not pre-treated with a matrix, spatial resolution is not compromised by matrix crystals, which could be larger than the studied cells.

Electrospray ionization (ESI) was originally designed to ionize long polymer chains (Dole et al., 1968) and has subsequently evolved (Yamashita and Fenn, 1984; Whitehouse et al., 1985) to a commonly used ion source in mass spectroscopy. ESI has become very popular (Bhardwaj and Hanley, 2014), for example, in combination with liquid chromatography (Whitehouse et al., 1985) and been used for MSI as well, especially in the form of desorption electrospray ionization (DESI; Bjarnholt et al., 2014) and the closely related nano-DESI (Lanekoff et al., 2012). These techniques have been shown to achieve 50 and 20 μm spatial resolution, respectively (Campbell et al., 2012; Lanekoff et al., 2012). Instead of extracting analytes prior to analysis, both techniques extract analytes *in situ* prior to ionization directly from the sample surface (Venter et al., 2014). Control over the amount of sample surfaces wetted becomes imperative to avoid cross contamination and maintain spatial resolution.

In 2007, LAESI was introduced (Nemes and Vertes, 2007). The basic principle of LAESI combines LDI and ESI: ablation with a laser, and ionization via ESI, as shown in **Figure 1C**. However, LAESI uses an IR laser and relies on water present in the sample as a makeshift matrix (Apitz and Vogel, 2005; Nemes et al., 2012), a condition that most samples in life sciences fulfill. This way the deposition of an external matrix is not required, sample handling is simplified and the need to manipulate the samples prior to analysis is reduced. In a LAESI source, IR-laser light of 2940 nm wavelength is used to irradiate samples. At this wavelength, water has a major peak in its absorption spectrum and thus acts as a chromophore absorbing the deposited energy (Hale and Querry, 1973; Downing and Williams, 1975). Essential work describing the physics of ablating biological tissue with a laser was done recently (Vogel and Venugopalan, 2003b). The event of sample ablation can be split into at least two different phases based on the tensile strength of the sample (Vogel and Venugopalan, 2003a; Apitz and Vogel, 2005). Initially, irradiated sample material is heated and vaporization of molecules from the surface takes place (Vogel and Venugopalan, 2003a). When the energy deposition of the laser is larger than the energy consumption of the vaporization process, the water content of the sample is further heated and driven into a superheated state, leading to phase explosion upon relaxation to a stable state (Vogel and Venugopalan, 2003a; Apitz and Vogel, 2005; Chen et al., 2006). This results in material expulsion as well as tissue rupture and is primarily responsible for ablation efficiency (Apitz and Vogel, 2005). The resulting ablation plume consists mostly of neutral matter in the form of nanoparticles, droplets, and large particulates. Experimental data suggest droplets from the electrospray plume intercept and fuse with the ablation plume nanoparticles, extracting analytes in the process (Nemes and Vertes, 2007). At this point, post-ionization by ESI takes over. A review of the research done on most of the aspects governing ESI (Kebarle and Verkerk, 2009) provides an excellent introduction to the field. Once ions have been generated from the sample, mass analyzers provide the means of detection.

The following section provides examples of instrumentation to illustrate the capabilities of the LAESI technique. LAESI displays promising potential for application in animal and plant metabolomics (Stolee et al., 2012; Stopka et al., 2014) and MSI of living plant tissue (Nemes and Vertes, 2007; Li et al., 2015b). For more information on different types of MSI methods, refer to **Table 1**.

**Table 1:** Ionization techniques used for mass spectrometry imaging (MSI) of biological samples.

Ionization technique	Typical spot size/spatial resolution	Requirements/sample preparation	Reference
Secondary ion mass spectrometry (SIMS)	~100 nm, subcellular resolution possible	Sample must be stable enough in vacuum environment	McMahon et al. (1995), Colliver et al. (1997), Mohr et al. (2013)
Matrix-assisted laser desorption/ionization (MALDI)	~10 μm with commercially available instruments	Matrix molecules need to be co-crystalized with sample	Karas and Kruger (2003), El-Baba et al. (2014)
Laser desorption/ionization (LDI)	~5 μm with commercially available instruments	UV-absorbing analytes increase desorption/ionization	Hölscher et al. (2009), Kroiss et al. (2010), Hoelscher et al. (2014)
Matrix-assisted laser desorption electrospray ionization (MALDESI)	Spot size is 250–300 μm, spatial resolution of 45 μm with oversampling reported	Similar to MALDI but higher ion yield achievable through post ionization step	Sampson et al. (2006), Robichaud et al. (2014)
Desorption electrospray ionization (DESI)	50–20 μm spatial resolution, depending on source instrumentation	No particular sample preparation needed but sensitive to surface wetting	Campbell et al. (2012), Lanekoff et al. (2012)
Laser ablation electrospray ionization (LAESI)	350–15 μm spot size, depending on source instrumentation	Water in sample, e.g., in the form of cytosol	Nemes and Vertes (2007), Shrestha and Vertes (2009)

## Application of LAESI

The first realization of a LAESI ion source, as described by Nemes and Vertes (2007), consisted of a custom-built electrospray system, an Er:YAG laser tuned to a wavelength of 2940 μm, and a time-of-flight (TOF) mass spectrometer. One of the proof-of-concept experiments carried out was metabolic profiling of *Tagetes patula* seedlings *in vivo*. Several tentative assignments of metabolites from roots, leaves and stems were made. For that, accurate mass measurements, isotope patterns and metabolomic databases of model organisms such as *Arabidopsis thaliana* were considered. Cautious use of these databases was justified under the presumption that plants share certain metabolomics features (Nemes and Vertes, 2007; Nemes et al., 2008). Although LAESI is classified as a destructive method, seedlings subjected to the single-shot laser ablation were reported to survive the 350 μm wide ablation craters in roots, leaves, and stems.

Nemes et al. (2008) used a combination of LAESI and TOF mass analyzer techniques to show the usability of LAESI for MSI of plant tissues. Leaves of *Aphelandra squarrosa* with variegation patterns were subjected to two-dimensional imaging with a spatial resolution of 400 μm and depth profiling with a resolution of 50 μm. The actual spot size of the laser was reported as 350 μm, but a bigger step size was chosen to limit cross-talk in the acquisition of mass spectra. Nemes et al. (2008) were able to show that localization of the secondary metabolites kaempferol and luteolin, as well as certain derivatives with sugar moieties, coincides with the variegation pattern. The spatial distribution was then combined with the information gathered from depth profiling to visualize the spatial distribution of secondary plant metabolites in three dimensions. Depth profiling was realized by consecutive irradiation of the same spot (Nemes et al., 2008, 2009).

The work of Nemes et al. (2008, 2009) showed the feasibility of a LAESI ion source for analyzing and imaging metabolites in plant samples. Shrestha and Vertes (2009) improved upon the LAESI concept by using an etched, GeO_2_-based glass fiber to focus and deliver the laser to the sample. This made it possible to decrease the diameter of the ablation marks to slightly larger than 2R, with R being the radius of the glass fiber tip’s curvature, reported as roughly 15 μm in size and as forming ablation craters of ca. 30 μm. The metabolome of single epithelial cells from *Allium cepa* and *Narcissus pseudonarcissus* bulbs was analyzed and compared across species, but also compared to relative species within a particular sample tissue. Interestingly, the same cell type, *A. cepa* bulb epithelial cells and their *N. pseudonarcissus* equivalent, showed different contents of metabolites, with oligosaccharides and alkaloid, respectively, abundant (Shrestha and Vertes, 2009). By looking at epithelia from different layers of the same bulb, differently aged *A. cepa* cells were compared. The content of arginine was reported to decrease with increasing cell age, while the alliin gradient was oriented the other way around. Cells in an *A. cepa* bulb are older when located in the outer layers. Shrestha et al. (2011) also determined the influence of ablating event on single cells within a tissue on the surrounding cells and found no major disturbance compared to similar cells in undisturbed areas of the sampled tissue.

The same experimental set-up was also used to find biomarkers in the oil glands of *Citrus aurantium* leaves. For the initial mass spectra from achlorophyllous cells of *C. auratium*, leaf oil glands and epidermal cells from distant parts of the same leaf were first measured and then compared. Different terpenes and terpenoids were found in the oil gland cells, which are absent in the epidermal cells and which contained flavonoids compounds not present in the gland cells (Shrestha et al., 2011).

The step to subcellular resolution was taken by Stolee et al. (2012). The LAESI set-up described previously (Shrestha and Vertes, 2009) was improved upon by adding a micro-dissection needle made out of tungsten. Prior to sample irradiation by the IR laser, the needle with a tip diameter of approximately 1 μm was used to cut open and peel back the cell wall of *A. cepa* epithelial cells. Metabolites such as hexose and alliin were reportedly found with higher abundance in cytosolic areas of a cell, whereas the amino acids arginine and glutamine were found more commonly in the area of the cell nucleus (Stolee et al., 2012). However, the improvement made by ablating the sample precisely goes hand in hand with the small sample volume from which ions can be generated. This limitation obviously reduces sensitivity of the method and poses a general problem of spatially confined ionization techniques.

Depending on the properties of the electrospray solution used, imaging substances with strongly diverging polarities may be difficult to ionize simultaneously. A LAESI source was modified to address this problem (Vaikkinen et al., 2013). By adding a nebulizer chip blowing heated nitrogen gas toward the MS orifice, a more efficient ionization of both polar and non-polar compounds was expected (Careri et al., 1999; Boscaro et al., 2002). Compared to an unmodified LAESI ion source, heat-assisted LAESI (HA-LAESI) has shown to better ionize compounds with low polarity, as demonstrated on *Persea americana* mesocarp (Vaikkinen et al., 2013). A high abundance of signals assigned to triglycerides was observed in the MS spectrum measured with HA-LAESI. These particular peaks were less pronounced when using LAESI. To demonstrate imaging capabilities, Vaikkinen et al. (2013) used *Viola* flower petals and visualized the distribution of glycosides known to be present in *Viola* (Saito et al., 1983) as shown in **Figure 1F**. To further improve on ionizing low and non-polar compounds, a krypton discharge lamp for photo-ionization was added to the LAESI set-up to ionize anisole molecules with UV light that in turn ionize analytes in subsequent reactions taking place in the gas phase. The electrospray was exchanged for a nebulizer chip with an anisole and heated nitrogen gas flow (Vaikkinen et al., 2014), very similar to HA-LAESI. The technique was called laser ablation atmospheric pressure photoionization (LAAPPI). MSI was performed on *Salvia officinalis* leaves, and tentative assignment of multiple terpene and terpenoid compounds could be made (Vaikkinen et al., 2014). Because the IR light was focused using a lens instead of an etched glass fiber (Shrestha and Vertes, 2009) as described by Nemes et al. (2008), spatial resolution was reported as 400 μm.

Until recently, MSI was performed by measuring a sample step-wise using a predefined raster. Resolution of the mapping thus depended on the smallest possible step preventing pixel cross-talk. Li et al. (2015b) reported a procedure for LAESI-MSI, integrating light microscopy to assess and identify single cells within a sample tissue. An imaging raster consisting of cells defining that particular sample tissue was then created and used for systematic cell-by-cell imaging. Feasibility and proof-of-concept experiments on *A. cepa* bulb and *Lilium longiflorum* were performed using the precision of LAESI with an etched, GeO_2_-based glass fiber (Shrestha and Vertes, 2009). The capacity for separating isobaric and structurally isomeric ions in LAESI-MSI experiments was demonstrated by Li et al. (2015a) on *Pelargonium peltatum* leaves and mouse brain tissue.

Trying to make LAESI more compatible with complementary methods such as light microscopy, Compton et al. (2015) tried to spatially separate laser ablation from ESI. After ablation, the produced plume was carried into transfer tubing with nitrogen gas, and analytes were ionized with ESI after emerging from the 60 cm long tubing. Parts of *Viola* and *Acer* sp. were analyzed using remote-LAESI as proof-of-principle experiments. Signal strength was reported to be 27% of the intensity detected using conventional LAESI (Compton et al., 2015).

Laser ablation electrospray ionization was recently used as one of the methods to confirm the quantitative MSI of surface-occurring glucosinolate on *A. thaliana* leaf surfaces (Shroff et al., 2015). Data obtained from LAESI and liquid extraction surface analysis (LESA; Kertesz and Van Berkel, 2010) unambiguously supported the data obtained using a 9-aminacridine matrix sublimed on the leaves and imaged using vacuum MALDI-MSI.

In addition, LAESI has been applied to human- and animal-derived samples. The applicability of LAESI to blood and serum samples for medical purposes as well as antihistamine quantification directly from human urine samples has been shown (Nemes and Vertes, 2007). Since then, metabolomic and lipidomic analysis of the electric organ of *Torpedo californica* (Sripadi et al., 2009), rat and mouse brain (Nemes et al., 2010; Shrestha et al., 2010), fish gills (Shrestha et al., 2013), and other samples (Parsiegla et al., 2012; Shrestha et al., 2014) has been reported. A LAESI system, DP-1000 LAESI, is now available commercially from Protea Bioscience (Morgantown, WV, USA). The spatial resolution of the system is ca. 200 μm and can be attached to diverse mass spectrometers. Early data on MSI of pesticides, mycotoxines, and plant metabolites from lemon or rose leaves have recently been published (Nielen and van Beek, 2014) using this source.

## Application of LDI-MSI *in Planta*

Laser desorption ionization can be applied *in planta*, as many important secondary metabolites contain conjugated double-bond systems like aromatic/heteroaromatic rings and show strong UV adsorption at 337 or 355 nm; both levels are emitted by the most common UV lasers. Plant pigments and compounds of the polyketide family readily absorb UV light and serve to desorb/ionize themselves. Elimination of MALDI matrices makes MSI in cellular resolution possible; see, for example, hypercins in glandular pigment cells of *Hypericum perforatum* or quercetin glucosides in *A. thaliana* petals or sepals as demonstrated by Hölscher et al. (2009) and shown in **Figure 1E**. A vacuum MALDI system Ultraflex (Bruker) with smart beam technology provided 10 μm spatial resolutions. Hypercins were shown to co-localize with dark pigment glands. A recent advance in developing systems with even higher spatial resolution as well as mass accuracy was commercialized in the AP-SMALDI imagine10 (TransMIT, Giessen, Germany) source attached to a Q-Exactive system with orbital mass analyzer (Thermo Scientific, San Jose, CA, USA). Laser spot sizes smaller than 5 μm are possible, and LDI measurements can be performed at ambient conditions thus preventing plant sample desiccation and deformation. This method is not limited to plants as was documented by MSIs of nematodes ingesting plant toxins from infected banana roots (Hoelscher et al., 2014) or on various MSI of antibiotics produced by actinomycetes on beewolf cocoons (Kroiss et al., 2010). LDI coupled with a plasma torch, also known as laser ablation inductively coupled plasma MS (LA-ICP-MS), is used for imaging distribution of metals *in planta* (Becker et al., 2010) or to localize proteins labeled with antibodies containing a metal-reporter ion (Bendall et al., 2011). This method shows extreme sensitivity, and as desorbed tissue debris undergoes post-ionization in a plasma torch, the technique is also quantitative.

## Conclusion

Although plant tissues have been employed to characterize LAESI since the introduction of the technique in 2007, its application in plant metabolomics and MSI is still limited to proof-of-concept experiments, for example, with onion (*A. cepa*) bulbs. This limited use may be a result of the apparent dominance of MALDI applications in imaging with high spatial resolution and the initial barrier of acquiring a LAESI source, since instrumentation with high spatial resolution is not yet commercially available. Even custom-built realizations do not reach the benchmark resolutions reported for MALDI. Advantages such as the absence of an external matrix and the potential for direct correlation with microscopically gathered data through the means of software evaluation may, however, promote the use of LAESI over time. Interdisciplinary work, in particular, which is usually characterized by a wide variety of methods and thus depends on data correlation, might profit from these ionization techniques. As the literature reviewed here shows, the performance of the LAESI ion source is sufficient for utilization in larger studies of plant metabolomes, especially in MSI of target metabolites, and for answering current biological questions. The same can be said about LDI. It is less intrusive than MALDI, because it does not require an externally applied matrix. Additionally, the spatial resolution is not compromised by the matrix crystals, which could be larger than the studied cells. Typically, using diverse orthogonal methods can be fruitful and is of help in reducing experimental bias.

## Conflict of Interest Statement

The authors declare that the research was conducted in the absence of any commercial or financial relationships that could be construed as a potential conflict of interest.
